# Fluorescein staining of chloroplast starch granules in living plants

**DOI:** 10.1093/plphys/kiad528

**Published:** 2023-10-04

**Authors:** Shintaro Ichikawa, Momoko Sakata, Toru Oba, Yutaka Kodama

**Affiliations:** Center for Bioscience Research and Education, Utsunomiya University, Tochigi 321-8505, Japan; Graduate School of Regional Development and Creativity, Utsunomiya University, Tochigi 321-8505, Japan; Center for Bioscience Research and Education, Utsunomiya University, Tochigi 321-8505, Japan; United Graduate School of Agricultural Science, Tokyo University of Agriculture and Technology, Tokyo 183-8509, Japan; Graduate School of Regional Development and Creativity, Utsunomiya University, Tochigi 321-8505, Japan; Faculty of Engineering, Utsunomiya University, Tochigi 321-8585, Japan; Center for Bioscience Research and Education, Utsunomiya University, Tochigi 321-8505, Japan; Graduate School of Regional Development and Creativity, Utsunomiya University, Tochigi 321-8505, Japan; United Graduate School of Agricultural Science, Tokyo University of Agriculture and Technology, Tokyo 183-8509, Japan

## Abstract

Chloroplast starch granules (cpSGs) store energy harvested through photosynthesis in plants, and cpSG dynamics have important roles in plant energy metabolism and stress responses. To date, cpSGs have been visualized using several methods, such as iodine staining; however, no method can be used to specifically visualize cpSGs in living cells from various plant species. Here, we report a simple method to visualize cpSGs in living plant cells in various species by staining with fluorescein, a commonly used fluorescent dye. We show that fluorescein is taken up into chloroplasts and interacts with cpSGs similarly to iodine. Fluorescein also interacts with refined starch in vitro. Using a fluorescein derivative for ultrabright cpSG imaging, we produced high-quality 3D reconstructions of cpSGs and evaluated their accumulation in multiple plant species. As fluorescein is well known and readily purchasable, our fluorescein-based staining method should contribute to all research regarding starch.

## Teaser

Fluorescein stains chloroplast starch granules in living plant cells and refined starch in vitro by entering the amylose and amylopectin vacancy in a similar way as iodine.

## Introduction

Chloroplast starch granules (cpSGs) store energy in plants. During the day, ADP-glucose derived from photosynthesis is polymerized into starch. Several cpSGs form in the stromal region of chloroplasts; for example, in Arabidopsis (*Arabidopsis thaliana*), 5 to 7 cpSGs are formed in each chloroplast (Crumpton-Taylor et al. 2012). These cpSGs are degraded into soluble carbohydrates at night for use in a variety of metabolic responses in source and sink tissues (Geiger et al. 2000; Smith and Stitt 2007; Stitt and Zeeman 2012; Yoon et al. 2021). Indeed, Arabidopsis mutants that do not form cpSGs exhibit inferior growth under day/night cycles compared to their wild type (Caspar et al. 1985; Gibon et al. 2004). The degradation of cpSGs mediates osmotic stress tolerance in plant cells (Thalmann et al. 2016) and causes stomatal opening in guard cells (Horrer et al. 2016); therefore, cpSGs are important polymers for plant growth and stress responses.

Over the past 2 centuries, several staining methods have been developed to visualize cpSGs, of which iodine staining is the oldest and most widely used (Colin and Gaultier de Claubry 1814; Abt et al. 2020). For iodine staining, decolorized leaves are stained with Lugol's solution containing iodine and potassium iodide. The iodine forms a complex with the long helical structure of amylose and the short helical structure of amylopectin, resulting in a purple coloration (Moulay 2013). Other methods of cpSG staining include toluidine blue and periodic acid Schiff (PAS) reagent, both of which can be visualized using light microscopy (Feder and O’Brien 1968; Abt et al. 2020). The above 3 staining methods can be used in various plant species but cannot be applied to living plant cells. For fluorescence imaging, modified pseudo-Schiff propidium iodide (mPS-PI) (Truernit et al. 2008) and safranin O staining (Liu et al. 2021) have been used in fixed and living cells, respectively; however, both fluorescent staining methods have low specificity for visualizing cpSGs because staining also targets other subcellular compartments, such as the vacuole. The NegFluo method was also developed by combining negative confocal imaging of chlorophyll autofluorescence with machine learning–based analysis, but this method needs chemically fixed cells (Vandromme et al. 2019). For specific fluorescence imaging in living cells, genetically encoded fluorescent proteins fused to cpSG-localized proteins, such as GRANULE-BOUND STARCH SYNTHASE I (GBSSI), can be used as cpSG markers (Szydlowski et al. 2009). However, the use of such cpSG markers requires genetic transformation, making them only amenable for use in limited plant species. To date, no method has been developed for specifically observing cpSGs in living cells of various plant species.

Here, we demonstrate that cpSGs can be fluorescently visualized by staining with fluorescein (Supplemental Fig. S1A), a xanthene-based fluorescent dye, in various living plant species. Fluorescein is one of the most widespread fluorescent dyes on the market. The staining method requires the simple immersion of a leaf disc in fluorescein solution for mere minutes. Fluorescein and its derivatives are, therefore, convenient and effective reagents for cpSG visualization in living plant cells.

## Results

### Live-cell cpSG staining using fluorescein

**Figure 1.**
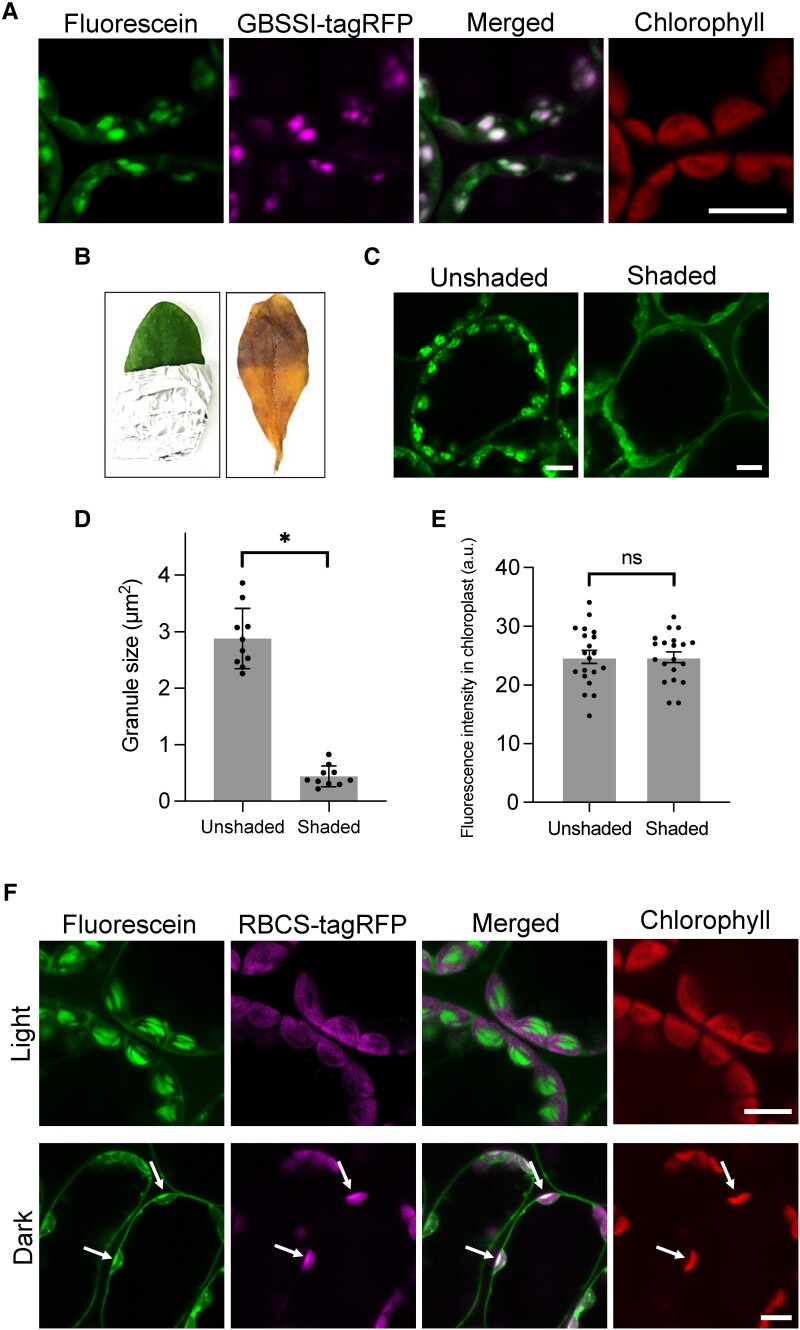
Fluorescein visualizes cpSGs in living plant cells. **A)** Leaves from transgenic *A. thaliana* plants expressing *GBSSI-tagRFP* observed using confocal microscopy after a 10 *µ*M fluorescein treatment. Scale bar, 10 *µ*m. **B)** Unshaded and shaded regions of an Arabidopsis leaf stained with Lugol's solution. Images were digitally extracted for comparison. **C)** Unshaded and shaded regions of an Arabidopsis leaf stained with 10 *µ*M fluorescein. Scale bars, 10 *µ*m. **D)** Sizes of fluorescent granules in the unshaded and shaded regions. An asterisk indicates a significant difference, as determined using Student's *t* test (*P* < 0.01). Values are means ± Sd (*n* = 10). **E)** Fluorescence intensity of fluorescein within the chloroplasts of unshaded and shaded leaf regions. There was no significant difference (ns) between the regions, as determined using Student's *t* test (*P* > 0.99). Values are means ± Sd (*n* = 20). **F)** Leaves of transgenic Arabidopsis plants expressing *RBCS-tagRFP* incubated under light (50 *µ*mol photons/m^2^/s) and dark conditions and then stained with 10 *µ*M fluorescein. Arrows indicate the merged region of fluorescein and RBCS-tagRFP signal in the stroma. Scale bars, 10 *µ*m.

We previously reported that a commercially available rhodamine B can be used to specifically visualize the chloroplast outer envelope membrane in living plant cells (Ichikawa et al. 2022). Given that the commercial fluorescent dye was applicable to observing the subcellular compartment, we treated Arabidopsis leaves with several other commercially available fluorescent dyes, seeking one that would mark subcellular structures. When we treated Arabidopsis leaves with fluorescein, the resulting fluorescent signal stained granular structures in the mesophyll, guard, and root tip cells (Supplemental Fig. S1, A and B). A time-lapse observation showed that fluorescein is transported into chloroplasts and allows the live-cell visualization of granular structures with chloroplast migration (Supplemental Movie S1). During transport to the organelle, fluorescein also produced signals in the cytosol (Supplemental Fig. S1C and Movie S1). Fluorescent granular structures could be observed with fluorescein incubations until 6 h, and then fluorescein transported to vacuoles with incubations for 12 h or more (Supplemental Fig. S2). Because the fluorescein-stained granular structure did not colocalize with chlorophyll autofluorescence (Fig. 1A), we hypothesized that fluorescein might be useful to visualize cpSGs. To assess whether fluorescein localizes to cpSGs, we expressed a genetically encoded cpSG marker, *GBSSI-tagRFP*, encoding GBBSI fused to the red fluorescent protein, in transgenic Arabidopsis plants and stained them with fluorescein. The fluorescein signal colocalized with the GBSSI-tagRFP signal (Fig. 1A), indicating that fluorescein localizes to cpSGs. To confirm that fluorescein can be used to visualize cpSGs, we stained the leaves of the Arabidopsis mutants *ss4* (defective in STARCH SYNTHASE 4) and *aps1* (lacking ADP-GLUCOSE PYROPHOSPHORYLASE SMALL SUBUNIT 1 function), which have abnormal cpSG numbers and morphologies, with fluorescein (Roldán et al. 2007; Bahaji et al. 2011; Crumpton-Taylor et al. 2013). Consistent with the previous studies, we detected 1 or 2 globular and large cpSGs in *ss4* and a few globular and tiny cpSGs in *aps1* (Supplemental Fig. S3). We, therefore, conclude that fluorescein can be used to visualize cpSGs in living cells of Arabidopsis.

### Fluorescein binds to cpSGs in the chloroplast stroma

To understand whether fluorescein staining can accurately reflect the presence of cpSGs, we shaded half of an Arabidopsis leaf with aluminum foil for 1 d to reduce the number of granules. We confirmed starch degradation in the shaded region by iodine staining (Fig. 1B). When the unshaded and shaded regions were treated with fluorescein, we observed almost no cpSGs in the shaded region (Fig. 1C), with those remaining being significantly smaller than cpSGs in the unshaded region (Fig. 1D). The fluorescent area of cpSGs in fluorescein staining was correlated with the starch content measured biochemically (Supplemental Fig. S4), indicating that fluorescein quantitatively detects starch levels. However, the fluorescence intensity of fluorescein throughout the entire chloroplast showed no significant difference between the 2 regions (Fig. 1E), indicating that fluorescein accumulates in chloroplasts regardless of the existence of cpSGs.

Since cpSGs form in the stroma within the chloroplast, we used fluorescein to stain the leaf of a transgenic Arabidopsis plant harboring a transgene encoding tagRFP fused to the stroma targeting signal sequence of RIBULOSE BISPHOSPHATE CARBOXYLASE SMALL CHAIN 1A (RBCS-tagRFP) under light and dark conditions. The fluorescein signal colocalized with that of RBCS-tagRFP in the stroma in the dark but not in the light (Fig. 1F). These data indicate that fluorescein interacts with cpSGs after its uptake into the stroma.

### Fluorescein binds to amylose and amylopectin helices

**Figure 2.**
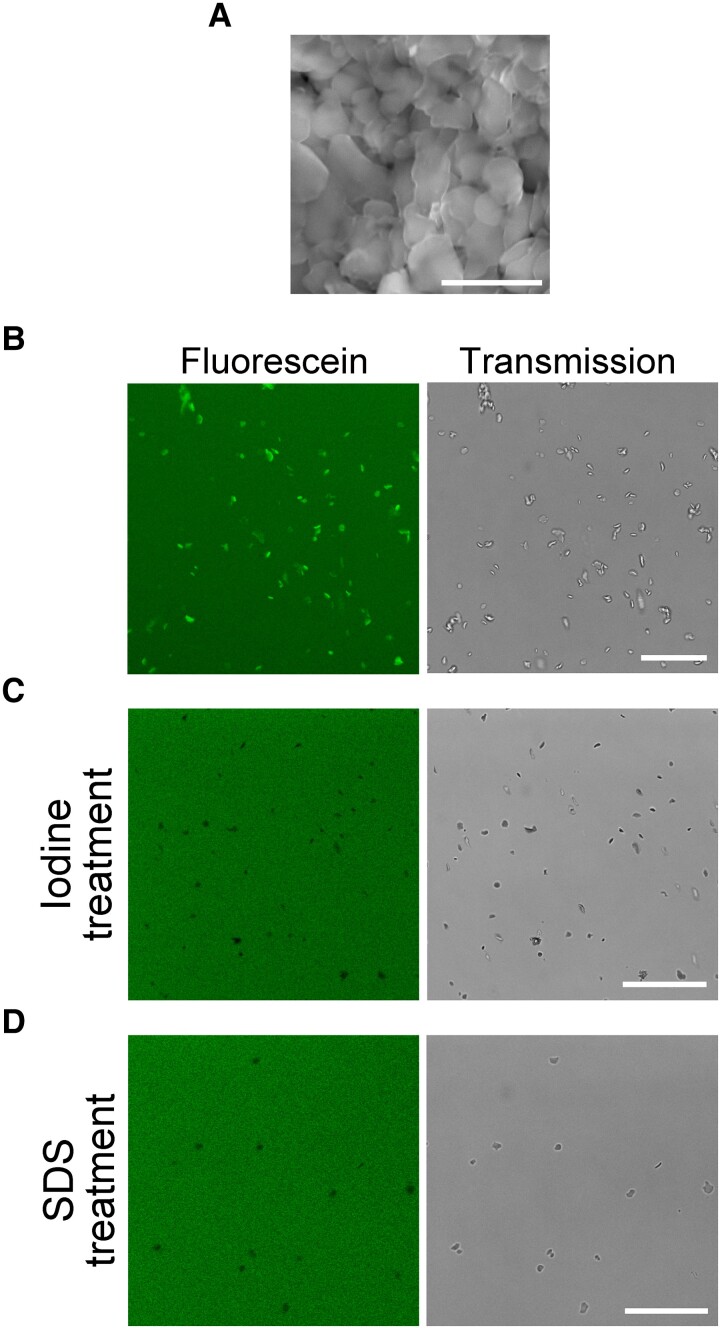
Fluorescein stains cpSGs via its interaction with the amylose helix. **A)** Isolated cpSGs observed using SEM. Stacked cpSG images were captured. Scale bar, 5 *µ*m. **B)** Isolated cpSGs stained with fluorescein in vitro. Scale bar, 25 *µ*m. **C, D)** Iodine-treated **C)** or SDS-treated **D)** cpSGs with fluorescein. Scale bars, 25 *µ*m.

To understand the interaction of fluorescein with cpSGs, we isolated cpSGs from Arabidopsis leaves and tested whether fluorescein directly binds to them. We confirmed the successful isolation of cpSGs by observation using scanning electron microscopy (SEM) (Fig. 2A). When the samples were stained with fluorescein, we observed fluorescence of the isolated cpSGs (Fig. 2B), indicating the interaction between fluorescein and cpSGs in vitro. A previous study reported that, in an anhydrous ethanolic solution, fluorescein enters into the amylose helix cavity of refined starch extracted from potato (*Solanum tuberosum*) (Tomasik and Zawadzki 1996), and we found that fluorescein also binds to refined rice (*Oryza sativa*), wheat (*Triticum aestivum*), and potato starch in aqueous condition and glycogen in ethanolic condition (Supplemental Fig. S5 and Movies S2 to S5). Based on this information, we speculated that fluorescein might interact with the amylose helix of cpSGs. To test this hypothesis, we used iodine, which mainly interacts with the amylose helix of starch, as a competitor for fluorescein (Rundle et al. 1944; Moulay 2013). When fluorescein was added to iodine-stained cpSGs, it did not visualize the cpSGs (Fig. 2C), suggesting that the iodine stain blocked fluorescein from interacting with the amylose helix of cpSGs. Because SDS also forms an inclusion complex with starch due to the insertion of the long-chain structure of SDS into the amylose helix (Yamamoto et al. 1983; Svensson et al. 1996), we also tested whether SDS-treated cpSGs might be stained with fluorescein. As with iodine-stained cpSGs, fluorescein failed to stain SDS-treated cpSGs (Fig. 2D).

Starch consists of amylose and amylopectin (Tester et al. 2004), and the results of the competition assays (Fig. 2, C and D) suggested that fluorescein can bind to the amylose helix of cpSGs. To test whether fluorescein also interacts with amylopectin, we treated the GBSSI-deficient mutant (*gbssI*) leaf that produces amylopectin-only (amylose-free) cpSGs with fluorescein (Shure et al. 1983), which revealed that these cpSGs can be visualized by fluorescein (Supplemental Fig. S6, A and B). In iodine staining, amylose-rich and amylose-less starch can be distinguished by color: amylose in blue and amylopectin in red (Moulay 2013). Although we attempted to distinguish the different types of starch stained with fluorescein, we found that the spectra of the excitation and emission of fluorescein interacting with the starch were similar between wild-type (amylose-rich) and *gbssI* mutant (amylose-free) Arabidopsis plants (Supplemental Fig. S6C). This suggests that the amylose content within starch cannot be determined using fluorescein staining. However, unlike iodine staining, fluorescein stained the heat-treated gelatinized starch in which the long helical structure of amylose and short helical structure of amylopectin are unfolded (Supplemental Fig. S7) (Svihus et al. 2005). This suggests that fluorescein can bind to a partially unfolded helical structure within starch.

Taken together, these findings show that fluorescein interacts with amylose and amylopectin in the cpSGs via the helical structures of these sugar polymers.

### cpSG imaging in various plant species

**Figure 3.**
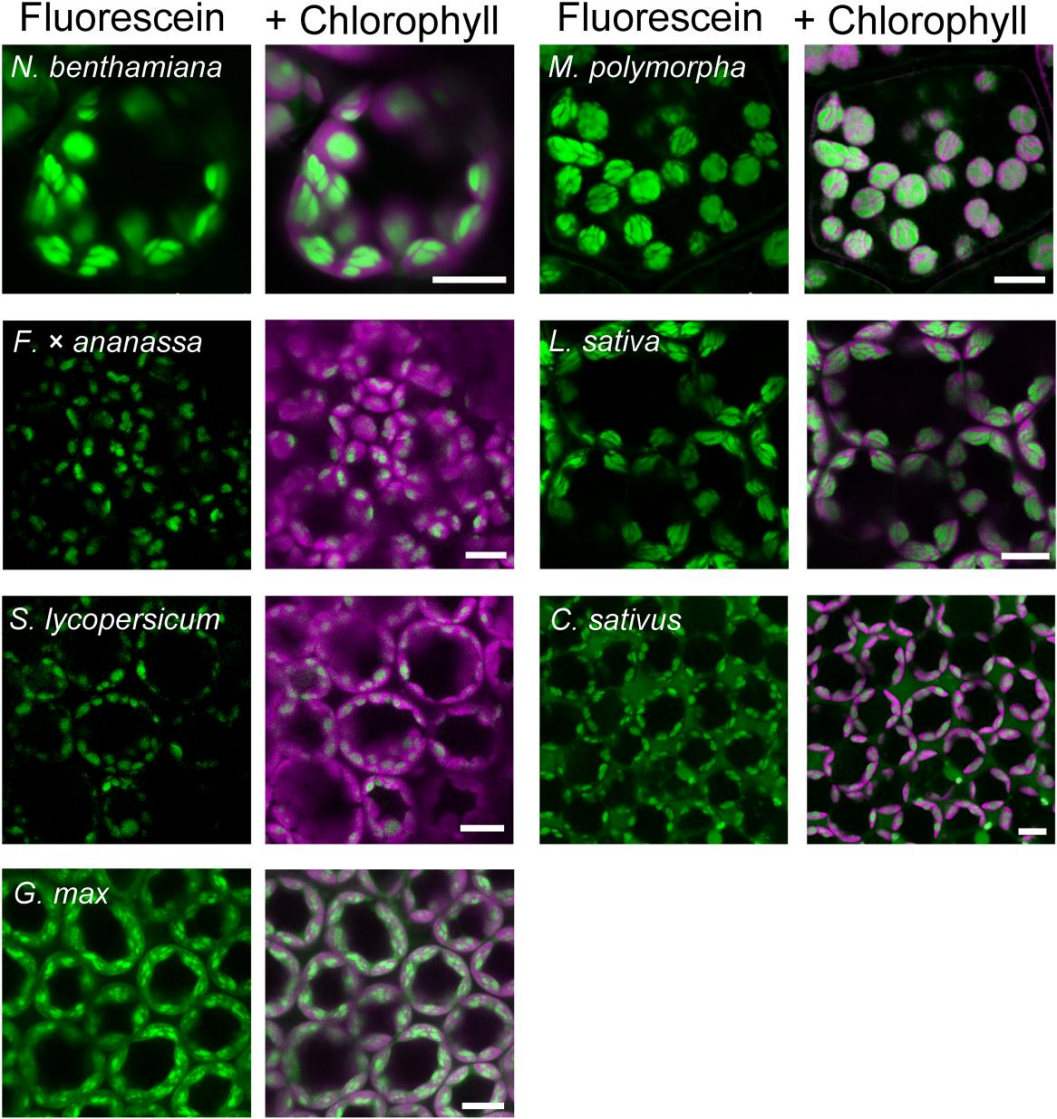
Fluorescein allows the visualization of cpSGs in various plant species. Representative images of cpSGs from *N. benthamiana*, liverwort (*M. polymorpha*), strawberry (*F. × ananassa*), lettuce (*L. sativa*), tomato (*S. lycopersicum*), cucumber (*C. sativus*), and soybean (*G. max*) stained with 10 *µ*M fluorescein. Scale bars, 10 *µ*m.

Live-cell imaging using fluorescent dyes can be performed on a wide range of species. We established that fluorescein staining for 10 min can be used to visualize cpSGs in multiple plant species, such as *Nicotiana benthamiana*, liverwort (*Marchantia polymorpha*), strawberry (*Fragaria × ananassa*), lettuce (*Lactuca sativa*), tomato (*Solanum lycopersicum*), cucumber (*Cucumis sativus*), and soybean (*Glycine max*) (Fig. 3). The cpSG imaging method using fluorescein is thus a facile and rapid technique applicable to a variety of plant species.

### 3D evaluation of cpSGs in living cells

**Figure 4.**
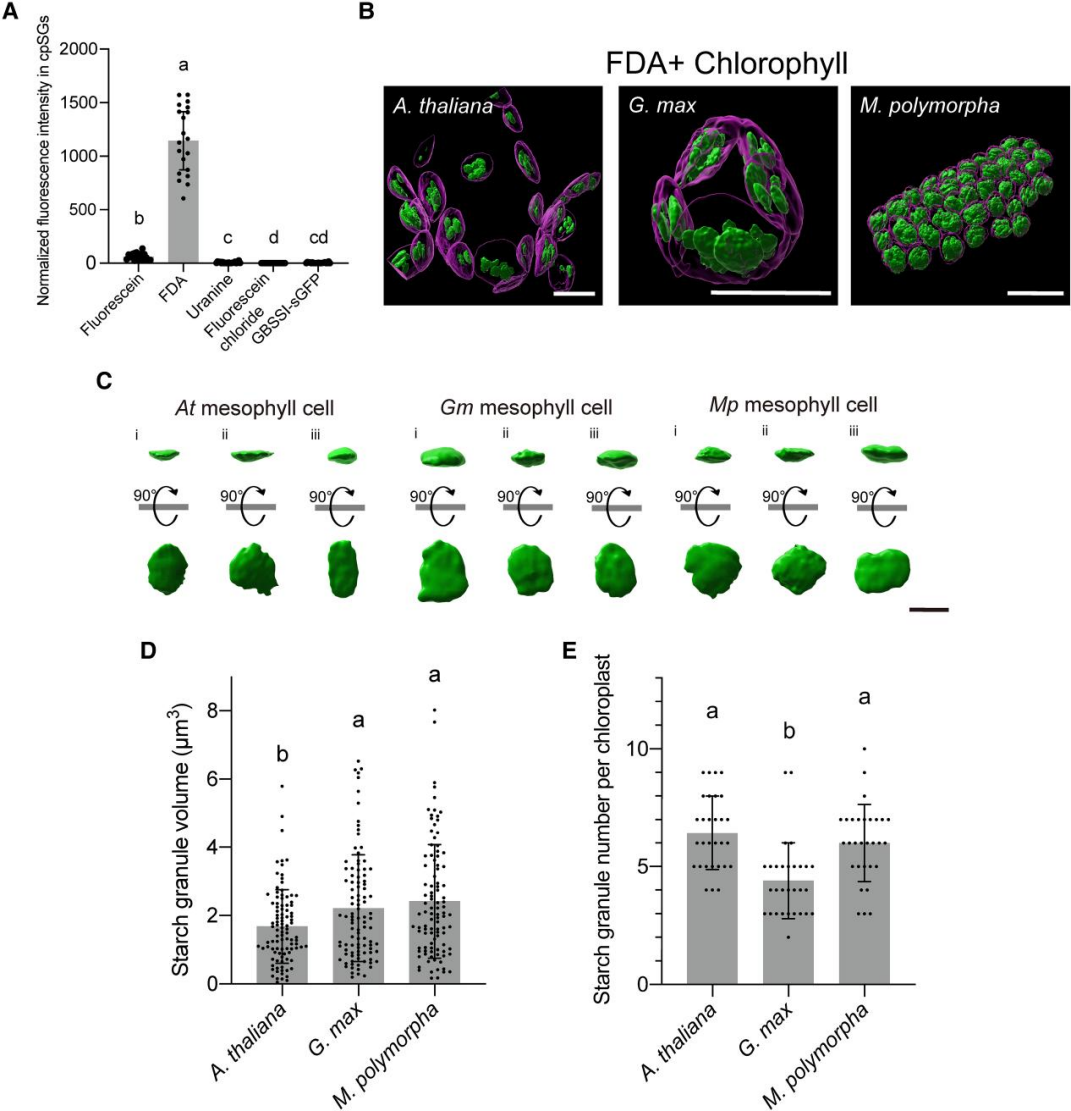
3D analysis of cpSGs in living cells using FDA. **A)** Fluorescence intensity of fluorescein, its derivatives (FDA, uranine, and fluorescein chloride), and GBSSI-GFP in cpSGs. Individual values were normalized using the median of fluorescence intensity from GBSSI-GFP. Different letters indicate significant differences, as determined using a Kruskal–Wallis and Steel–Dwass test (*P* < 0.01). Values are medians ± 95% confidence interval (*n* = 20). **B)** 3D reconstructions of cpSGs and chloroplasts from Arabidopsis, *G. max*, and *M. polymorpha* obtained using FDA staining. Scale bars, 10 *µ*m. **C)** 3D reconstructions of individual cpSGs in Arabidopsis, *G. max*, and *M. polymorpha*. Reconstructions captured from the *x*–*y* plane and 90°-rotated reconstructions are shown. Scale bar, 2 *µ*m. **D, E)** Volume of each cpSG **D)** and number of cpSGs per chloroplast **E)** in Arabidopsis, *G. max*, and *M. polymorpha*. Different letters indicate significant differences, as determined using a 1-way ANOVA and Tukey's multiple comparison test (*P* < 0.05). Values are means ± Sd (**C**, *n* = 100; **D**, *n* = 30).

We discovered that several fluorescein derivatives, such as fluorescein diacetate (FDA), uranine, and fluorescein chloride, can also be used to visualize cpSGs in planta (Supplemental Fig. S8). Among these derivatives, the fluorescence intensity of FDA in cpSGs was much greater than that of fluorescein (Fig. 4A; Supplemental Fig. S9A). The fluorescence intensity of FDA in wild-type Arabidopsis leaves was 1,144-fold brighter than that of GBSSI-sGFP in transgenic Arabidopsis leaves, in which the *GBSSI-sGFP* gene is driven by cauliflower mosaic virus (CaMV) 35S promoter to an excessive level (Fig. 4A; Supplemental Fig. S9B). FDA can, therefore, be used to visualize cpSGs in living cells with an ultrahigh fluorescence intensity. Because FDA can be excited by the very weak laser, the ultrahigh fluorescence intensity of FDA was maintained without photobleaching even under continuous laser irradiation for 30 min, in contrast to fluorescein and GBSSI-sGFP (Supplemental Fig. S10). FDA staining enables long-time observation of cpSGs, including applications such as time-lapse and 3D imaging.

Using FDA staining, we attempted to determine the volume of individual cpSGs and the cpSG number per chloroplast in living Arabidopsis mesophyll cells. Accordingly, we captured 3D confocal cpSG and chlorophyll (thylakoid) images. The captured raw images were deconvoluted to improve contrast and resolution, providing a clearer outline of cpSGs and thylakoids. We then reconstructed the deconvoluted images into 3D images, with which we evaluated individual cpSGs (Fig. 4, B to E; Supplemental Movie S6). The morphology of cpSGs resembled that of the isolated cpSGs observed using SEM (Figs. 2A and 4C). In mesophyll cells, the cpSG volume was 1.68 ± 1.07 *µ*m^3^ (Fig. 4D); we counted 6.43 ± 1.54 cpSGs per chloroplast (Fig. 4E). These live-cell data are consistent with the findings of a previous study based on fixed cells using focused ion beam SEM (FIB-SEM) (Crumpton-Taylor et al. 2012). Thus, the accuracy of FDA-based confocal microscopy for cpSG imaging in living cells is comparable to that of FIB-SEM in fixed cells.

We also produced 3D reconstructions of cpSGs in the other plant species, *G. max* and *M. polymorpha*. The cpSG volumes were 2.22 ± 1.55 *µ*m^3^ in *G. max* and 2.42 ± 1.65 *µ*m^3^ in *M. polymorpha* (Fig. 4D), both of which were larger than the measured volumes for Arabidopsis. The visualized chloroplasts contained 4.40 ± 1.58 cpSGs in *G. max* and 6.00 ± 1.61 in *M. polymorpha* (Fig. 4E), indicating that the chloroplasts of *G. max* contain fewer cpSGs than those of Arabidopsis and *M. polymorpha*. The FDA-based cpSG imaging successfully parameterized the steric information of these granules in living cells of different plant species, the comparison of which suggests that the formation of cpSGs differs among plant species.

## Discussion

Several methods have been reported for staining starch and cpSGs using dyes. The iodine staining technique was developed over 200 years ago and is still widely used today. Iodine mainly binds to the long helix of amylose and to the short helix of amylopectin in starch. In toluidine blue staining, starch is stained by metachromasia, which changes color by binding toluidine blue (a cationic dye) to negatively charged starch (Sridharan and Shankar 2012). The present study suggests that fluorescein interacts with the helical structures of amylose and amylopectin in cpSGs and allows the visualization of their morphology (e.g. Fig. 1A; Supplemental Fig. S6B). Fluorescein is an anionic dye (Supplemental Fig. S1A); thus, the molecular mechanism of fluorescein staining is similar to that of iodine staining but not toluidine blue staining. Since chloroplasts in the fluorescein-treated cells were migrating (Supplemental Movie S1), unlike iodine staining, fluorescein can be used in living cells.

Similar to iodine, fluorescein could stain both starch and glycogen (Supplemental Fig. S5) (Moulay 2013). Iodine staining is also known to differentiate between amylose and amylopectin within starch by color, but it cannot stain gelatinized starch (Moulay 2013). On the other hand, fluorescein was not able to differentiate between amylose and amylopectin within starch (Supplemental Fig. S6C), but it could stain gelatinized starch (Supplemental Fig. S7) (Moulay 2013). For iodine-based coloration of starch, iodine molecules require sequential linkage with each other within the helical structure of amylose and amylopectin (Moulay 2013). Conversely, fluorescein molecules can emit fluorescence without their molecular association. Therefore, it is suggested that fluorescein stained the gelatinized starch, where the partially unfolded helical structure may still be present (Supplemental Fig. S7). While the starch staining mechanism using fluorescein is similar to that using iodine, it is important to note that fluorescein staining cannot entirely replace iodine staining. The selection of these staining methods should be based on the experimental purpose.

The underlying mechanisms for the transport of fluorescein into chloroplasts remain unknown but would require permeation through the plasma membrane and the chloroplast envelope membranes (outer and inner). A previous study reported that fluorescein is not membrane permeable (Pfautsch et al. 2015); therefore, it must be taken up into plant cells via unknown transport pathways. Time-lapse observations in Arabidopsis cells after treatment with fluorescein revealed that it initially accumulated in the cytosolic region before moving into chloroplasts (Supplemental Fig. S1C). A fluorescein derivative, FDA, is a plasma membrane–permeant dye; after FDA penetrates through the plasma membrane, fluorescein is generated from FDA in the cytosol due to its hydrolysis by cytosolic esterases (Widholm 1972). Importantly, when we used FDA, fluorescein generated in the cytosol was transported into chloroplasts. In this context, fluorescein transport pathways may be independent of the plasma membrane and the chloroplast envelope membranes. Elucidating the molecular mechanisms facilitating fluorescein transport in plant cells is, therefore, our next challenge.

Among fluorescein and its derivatives, FDA showed the highest fluorescence intensity at cpSGs. In plant cells treated with membrane-permeant FDA, fluorescein seems to highly accumulate compared to the other dyes, which may explain why cpSGs show much brighter fluorescence in cells treated with FDA than fluorescein (Fig. 4A). We also revealed that the fluorescence intensity at cpSGs in wild-type cells stained with FDA was much higher than that in GBSSI-sGFP expressing transgenic cells (Fig. 4A). Therefore, FDA would visualize cpSGs brighter than genetically encoded fluorescent proteins. Importantly, FDA and fluorescein staining can be used for colocalization analysis with starch-related proteins tagged with fluorescent proteins in living cells.

In the present study, we successfully stained cpSGs in model plants (Arabidopsis, *N. benthamiana*, and *M. polymorpha*) and crops (*F. × ananassa*, *L. sativa*, *S. lycopersicum*, *C. sativus*, and *G. max*). Because we were able to stain both an early-divergent land plant (*M. polymorpha*) and flowering plants (e.g. Arabidopsis), it is likely that the cpSGs of almost all plant species can be stained using fluorescein. Fluorescein-based cpSG staining will enable the comparison of cpSGs among various plant species. Indeed, the present study showed that the number, volume, and morphology of cpSGs vary among the tested plant species (Figs. 3 and 4, B to E). The measurement accuracy of our FDA-based method was comparable to that of FIB-SEM (Fig. 4, D and E) (Crumpton-Taylor et al. 2012). However, in a previous study, serial block-face SEM (SBF-SEM) detected tiny starch granules (about 80 nm in diameter), and the number of cpSGs per chloroplast was counted to be 12.8 ± 2.3 in Arabidopsis (Bürgy et al. 2021). The number obtained in SBF-SEM is higher than that in our fluorescein-based imaging (Fig. 4E). Fluorescein-based imaging cannot detect tiny cpSGs due to the limit of resolution of confocal microscopy. By combining fluorescein-based cpSG staining with mutant screenings and genetic analyses, it might be possible to discover the factor(s) underlying cpSG formation in a wide range of plant species.

Fluorescein-based cpSG staining can be used in planta and in vitro, and the method is also applicable for refined starch and glycogen. Moreover, fluorescein is one of the most widely used fluorescent dyes, and both fluorescein and its derivatives are commercially available. Taking into account the simplicity, specificity, versatility, and accessibility of our method, fluorescein-based cpSG staining has the potential to catalyze groundbreaking innovation in various research fields regarding cpSGs and starch, including basic plant science, agricultural science, and food science.

## Materials and methods

### Plant materials and growth conditions

Arabidopsis (*A. thaliana*) (Columbia-0 accession) was grown on half-strength MS medium containing 0.5% (w/v) gellan gum and 1% (w/v) sucrose for 12 d under constant white light (approximately 50 *µ*mol photons/m^2^/s) at 22 °C, before being transferred to a soil mixture of vermiculite and potting mix (2:1 [v/v]). Soybean (*G. max*), lettuce (*L. sativa*), and cucumber (*C. sativus*) were grown on the same soil mixture under the same light conditions as Arabidopsis at 22 °C. The liverwort *M. polymorpha* (Takaragaike-1 strain) was grown on half-strength Gamborg's B5 medium containing 1% (w/v) agar under the same continuous white light at 22 °C. *N. benthamiana* plants were grown on the soil mixture in a 16-h light/8-h dark photoperiod with 50 *µ*mol photons/m^2^/s light intensity at 25 °C. Strawberry (*F. × ananassa*) and tomato (*S. lycopersicum*) were harvested in Sakura city, Tochigi, Japan. Three Arabidopsis mutants, lacking SS4 (At4g18240), APS1 (At5g48300), or GBSSI (At1g32900) function, were obtained from the Arabidopsis Biological Resource Center (ABRC). The *ss4* (GABI-290D11) and *aps1* (SALK_040155) mutants were previously characterized (Roldán et al. 2007; Azoulay-Shemer et al. 2018), and their mutations were confirmed by genotyping. The *gbssI* mutant (SAIL_742_E07C1) was not previously characterized; therefore, the null mutation of *GBSSI* in the *gbssI* mutant was confirmed by performing reverse transcription PCR (RT-PCR) using the primers 5′-AACCAATTCAGTCGACATGGCAACTGTGACTGCTA-3′ and 5′-AAGCTGGGTCTAGATATCCCGGCGTCGCTACGTTCTC-3′ for *GBSSI* detection and 5′-AGAGATTCAGATGCCCAGAAGTCTTGTTCC-3′ and 5′-AACGATTCCTGGACCTGCCTCATCATACTC-3′ for *ACTIN2* (*ACT2*; At3g18780) detection.

### Plasmid construction and transformation

The DNA fragment encoding *GBSSI* was PCR amplified from Arabidopsis genomic DNA using the primers 5′-AACCAATTCAGTCGACATGGCAACTGTGACTGCTA-3′ and 5′-AAGCTGGGTCTAGATATCCCGGCGTCGCTACGTTCTC-3′. The *GBSSI* fragment was cloned into the pENTR1A vector (Thermo Fisher Scientific) digested by *Sal*I and *Eco*RV using In-Fusion Cloning (Takara Bio). The resulting pENTR1A-GBSSI clone was recombined into pGWB560 and pGWB605 binary vectors to generate pGWB560-GBSSI and pGWB605-GBSSI using a Gateway LR reaction (Thermo Fisher Scientific). The pGWB560 and pGWB605 binary vectors are designed to express a cloned gene under the control of the CaMV *35S* promoter with a 3′ in-frame *tagRFP* or *sGFP* tag, respectively (Nakagawa et al. 2007). pDONR207-RBCS-tagRFP (Osaki and Kodama 2017) was recombined into the pGWB602 binary vector to generate pGWB602-RBCS-tagRFP using the Gateway LR reaction. The pGWB602 binary vector is designed to express a cloned gene under the control of the CaMV *35S* promoter (Nakagawa et al. 2007). The pGWB560-GBSSI and pGWB605-GBSSI plasmids were introduced into *Agrobacterium* (*Agrobacterium tumefaciens*) strain GV2260, and the pGWB602-RBCS-tagRFP plasmid was introduced into *Agrobacterium* strain GV3101::pMP90. The *Agrobacterium* transformants were used for genetic transformation of Arabidopsis using the floral dip method (Clough and Bent 1998). The GBSSI-sGFP protein contained in transgenic Arabidopsis was found to accumulate to approximately 0.0176% of total protein (Supplemental Fig. S11).

### Isolation of cpSGs from Arabidopsis

A 600-mg (fresh weight) sample of 30- to 40-d-old Arabidopsis leaves was homogenized in liquid nitrogen using a mortar and pestle, after which 1 mL of extraction buffer (20 mM HEPES-KOH, pH 7.5, 1% [v/v] Triton X-100) was added. The homogenate was filtered through 2 layers of Miracloth (Merck), and Percoll buffer (20 mM HEPES-KOH, pH 7.5, 50% [w/v] Percoll) was added to the filtrate. The mixture was centrifuged at 800 *× g* for 5 min at 4 °C. After removing the supernatant, washing buffer (20 mM HEPES-KOH, pH 7.5) was added to the pellet for resuspension; the suspension was centrifuged at 800 *× g* for 5 min at 4 °C. These washing procedures were repeated 3 times. The resulting pellet containing starch granules was air dried and preserved at −30 °C until use.

### Immunoblot analysis

Leaves from 2-wk-old Arabidopsis seedlings expressing *GBSSI-sGFP* were collected and homogenized in a mortar and pestle with protein extraction buffer (150 mM NaCl, 1% [v/v] Triton X-100, and 50 mM Tris-HCl, pH 8.0) containing a protease inhibitor mixture (cOmplete Mini; Roche). The homogenates were mixed with 2× sample buffer (125 mM Tris-HCl, pH 6.8, 4% [w/v] SDS, 10% [v/v] glycerol, 0.01% [w/v] bromophenol blue, and 10% [v/v] 2-mercaptoethanol) and centrifuged at 15,000 *× g* for 10 min at 4 °C. The total protein amount in the supernatant was determined using the Bradford assay (Bio-Rad Laboratories). The extracted protein and recombinant GFP (Takara Bio; catalog no. 632373) used for the standard curve were incubated at 95 °C for 5 min. Next, about 5 or 7 *µ*g of the extracted protein and 0.25, 0.5, 1.25, and 2.0 ng recombinant GFP were applied to a 12% (w/v) SDS–PAGE gel. After separation by SDS–PAGE, the electrophoresed proteins were transferred onto a PVDF membrane. GBSSI-sGFP and recombinant GFP were detected using a 5,000-fold diluted anti-GFP antibody (Roche; catalog no. 11814460001) as the primary antibody, and a 3,000-fold diluted horseradish peroxidase–jointed anti-mouse IgG antibody (Thermo Fisher Scientific; catalog no. 32430) was used as the secondary antibody. Chemiluminescence signal was detected using ECL-Select (GE Healthcare) and Light Capture (ATTO).

### Staining experiments

Leaves from 2- to 3-wk-old Arabidopsis plants were excised using a hole puncher (Natsume Seisakusho; KN-291-2), making 2.0-mm-diameter leaf discs. After deaeration with water, each leaf disc was stained for 10 min with 10 *µ*M fluorescent dye solution (fluorescein, FDA, uranine, or fluorescein chloride) dissolved in water. The CAS numbers are 2321-07-5 (fluorescein), 596-09-8 (FDA), 518-47-8 (uranine), and 630-88-6 (fluorescein chloride). Each stained leaf disc without rinsing was then observed using confocal microscopy. Leaf discs excised from 3-wk-old *N. benthamiana*, *G. max*, *C. sativus*, and *L. sativa* plants and harvested *F. × ananassa* and *S. lycopersicum* plants were stained with 10 *µ*M fluorescein in the same way as the Arabidopsis leaf discs. Roots from 20-d-old Arabidopsis plants were excised to obtain the root tips, deaerated, and stained with 10 *µ*M fluorescein using a vacuum chamber. One-day-old *M. polymorpha* gemmalings cultured on half-strength B5 agar medium were stained with 10 *µ*M fluorescein for 10 min.

Isolated cpSGs were stained with 1 *µ*M fluorescein dissolved in the same buffer (20 mM HEPES-KOH, pH 7.5) used to wash isolated cpSGs and then mounted on a slide glass in fluorescein solution. Rice (*O. sativa*), wheat (*T. aestivum*), and potato (*S. tuberosum*) starch (Wako 193-13215, Sigma-Aldrich S7260, and MPBio 102954, respectively) were stained with 1 *µ*M fluorescein in the buffer for 30 min before being observed. Glycogen (Sigma-Aldrich G0885) was stained with 20 *µ*M fluorescein in 100% ethanol for 30 min. For 3D visualization of potato starch, it was rinsed with the buffer once to remove the background fluorescence. Images were captured for rice starch, wheat starch, and glycogen without rinsing. In the competition experiments using iodine and SDS, cpSGs were treated with one-fourth diluted Lugol's solution (Millipore Sigma) or 2% (w/v) SDS in the buffer for 30 min. After adding 1 *µ*M fluorescein to iodine- or SDS-treated cpSGs, the suspended cpSGs were observed using confocal microscopy.

For iodine staining, a leaf from a 30- to 40-d-old Arabidopsis plant grown under continuous light was partly shaded with aluminum foil for 24 h, after which the leaf was decolored using 100% ethanol. The decolored leaf was rinsed with water to remove ethanol and immersed in 4-fold-diluted Lugol's solution in water for 30 min. The Lugol-stained leaf was rinsed twice with water and observed.

### Microscopy

Confocal microscopy was carried out using an SP8X confocal microscope system (Leica Microsystems) with a water objective lens (Leica Microsystems; HC PL APO 63×/1.20 W CORR CS2) and photon counting mode. As the basic condition, a white light laser (WLL) at 21% intensity was used for excitation, and images were captured at 512 × 512 pixels with a 98-*µ*m pinhole. To compare the obtained cpSG fluorescence intensities among the GBSSI-sGFP, fluorescein, and derivative treatments, the pinhole size was set to 49 *µ*m, and the WLL at 0.7% intensity was used for the excitation of GBSSI-sGFP and the fluorescent dyes. For the 3D visualization of cpSGs, images were captured at 1,024 × 1,024 pixels with a 49-µm pinhole, and the WLL at 0.7% intensity was used for the excitation of FDA. For the excitation of chlorophyll autofluorescence, the WLL at 14% intensity was used in the fluorescence comparison and cpSG 3D visualization experiments. Fluorescein, FDA, uranine, fluorescein chloride, and sGFP emissions were detected between 500 and 550 nm with a 488-nm excitation. tagRFP emission was detected between 570 and 630 nm with 555-nm excitation. Chlorophyll autofluorescence emissions were detected between 680 and 730 nm with an excitation at 488 nm (under basic conditions) or 600 nm (under the cpSG fluorescence comparison and 3D cpSG visualization conditions). To eliminate chlorophyll autofluorescence, the time-gating method was used in fluorescein, FDA, uranine, fluorescein chloride, sGFP, and tagRFP images with a gating time of 0.5 to 1.2 ns (Kodama 2016). SEM of isolated cpSGs was carried out using a Miniscope TM3030 (Hitachi High-tech).

### Starch content measurement in leaf tissue

Samples of 100 mg (fresh weight) rosette leaves illuminated with white light (approximately 50 *µ*mol photons/m^2^/s) at 22 °C for 0, 4, and 8 h after 24-h dark treatment were harvested from 30- to 40-d-old Arabidopsis. Harvested leaves were homogenized in liquid nitrogen using a mortar and pestle. The mixture of the homogenates and 5 mL of 80% (v/v) ethanol was incubated at 80 °C for 5 min. After adding 5 mL of 80% ethanol, the samples were centrifuged at 1,000 × *g* for 10 min at 20 °C, and the supernatant was removed. The pellet was suspended with 10 mL of 80% ethanol, and the resulting suspension was centrifuged at 1,000 × *g* for 10 min at 20 °C. After removing the supernatant, the following procedures were performed using a Starch Assay Kit (Sigma-Aldrich; catalog no. STA20).

### 3D analysis of cpSGs

Confocal images of FDA and chlorophyll autofluorescence for 3D visualization were deconvoluted with Huygens Essential software (Scientific Volume Imaging) using default settings. The voxel size was set to 50 × 50 × 207 nm. The deconvoluted images were loaded into Imaris software (v9.8.0; Bitplane) for morphological analysis of cpSGs. 3D reconstructions of cpSGs and chloroplasts were obtained from FDA and chlorophyll autofluorescence images, respectively, and the resulting cpSG graphics were extracted using the Surfaces tool. The volume of a cpSG was measured using the Statistics mode of the Surfaces tool. In the case of adhesion between cpSGs, the adhered granules were individually divided based on the original deconvoluted images using the Edit mode of the Surfaces tool. The cpSGs in each chloroplast were counted using the Spot tool and a visual estimation from the original deconvoluted images.

### Accession numbers

Sequence data from this article can be found in the GenBank/EMBL data libraries under accession numbers *GBSSI* (At1g32900), *SS4* (At4g18240), *APS1* (At5g48300), and *RBCS1A* (AT1G67090).

## Supplementary Material

kiad528_Supplementary_Data

## Data Availability

All data used in this study can be found within the manuscript and its expanded data.

## Supplemental data

The following materials are available in the online version of this article.

**Supplemental Figure S1.** Fluorescein allows the visualization of granular structures in chloroplasts.

**Supplemental Figure S2.** Prolonged treatment of fluorescein in Arabidopsis.

**Supplemental Figure S3.** Fluorescein allows the visualization of cpSGs with abnormal number and morphology.

**Supplemental Figure S4.** Fluorescence signals of fluorescein for cpSGs correlate with the starch content.

**Supplemental Figure S5.** Fluorescein stains refined starch.

**Supplemental Figure S6.** Fluorescein stains cpSGs in the *gbssI* mutant.

**Supplemental Figure S7.** Fluorescein stains gelatinized starch.

**Supplemental Figure S8.** Fluorescein derivatives allow the visualization of cpSGs.

**Supplemental Figure S9.** FDA is the brightest derivative.

**Supplemental Figure S10.** Photobleaching test using FDA, fluorescein, and GBSSI-sGFP.

**Supplemental Figure S11.** Immunoblotting to quantify the abundance of GBSSI-sGFP in transgenic Arabidopsis.

**Supplemental Movie S1.** The uptake of fluorescein into plant cells.

**Supplemental Movie S2.** The rotation movie of the 3D image of rice starch.

**Supplemental Movie S3.** The rotation movie of the 3D image of wheat starch.

**Supplemental Movie S4.** The rotation movie of the 3D image of potato starch.

**Supplemental Movie S5.** The rotation movie of the 3D image of glycogen.

**Supplemental Movie S6.** 3D illustration of cpSGs and thylakoids in Arabidopsis mesophyll cells.

## Dive Curated Terms

The following phenotypic, genotypic, and functional terms are of significance to the work described in this paper:

CAS Gramene: AT5G23060
CAS Araport: AT5G23060

APS1 Gramene: AT3G22890
APS1 Araport: AT3G22890

fluorescein CHEBI: CHEBI:31624

iodine Gramene: EO:0007547
iodine Araport: EO:0007547

starch CHEBI: CHEBI:28017

ACT2 Gramene: AT3G18780
ACT2 Araport: AT3G18780

RBCS1A Gramene: AT1G67090
RBCS1A Araport: AT1G67090

glycogen CHEBI: CHEBI:28087
