# Supplementary material for: Fluorescein staining of chloroplast starch granules in living plants
Source: Plant Physiol. 2023 Oct 4;194(2):662–72. doi: 10.1093/plphys/kiad528 (PMC10828193; doi:10.1093/plphys/kiad528)
Supplement: kiad528_Supplementary_Data [file kiad528_supplementary_data.zip › Supplemental Data.pdf]

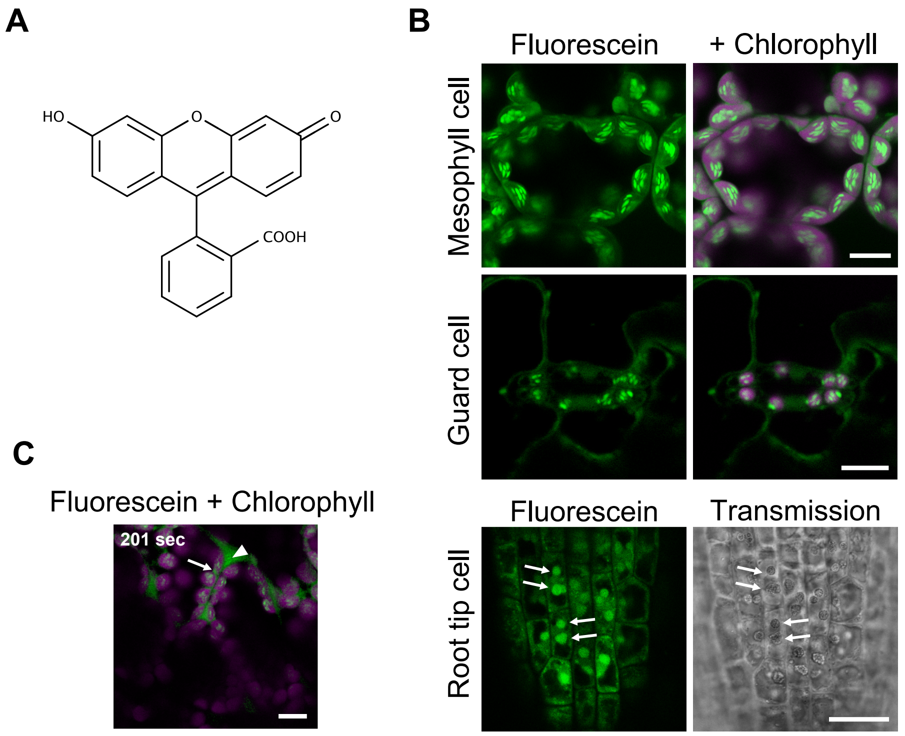

Supplemental Figure S1. Fluorescein allows the visualization of granular structures in chloroplasts.

A) Chemical structure of fluorescein. **B)** Granular structures visualized using fluorescein staining were observed in mesophyll, guard, and root tip cells of Arabidopsis using confocal microscopy. Arrows in the images of root tip cells indicate several granular structures observed in both fluorescence and transmission images. Scale bars, 10 µm. **C)** Fluorescein signal in both chloroplasts and the cytosol in a snapshot image at 201 s from Supplemental Movie S1. The arrow indicates the cytosolic signal of fluorescein, and the arrowhead indicates the apoplastic signal of fluorescein. Scale bar, 10 µm.

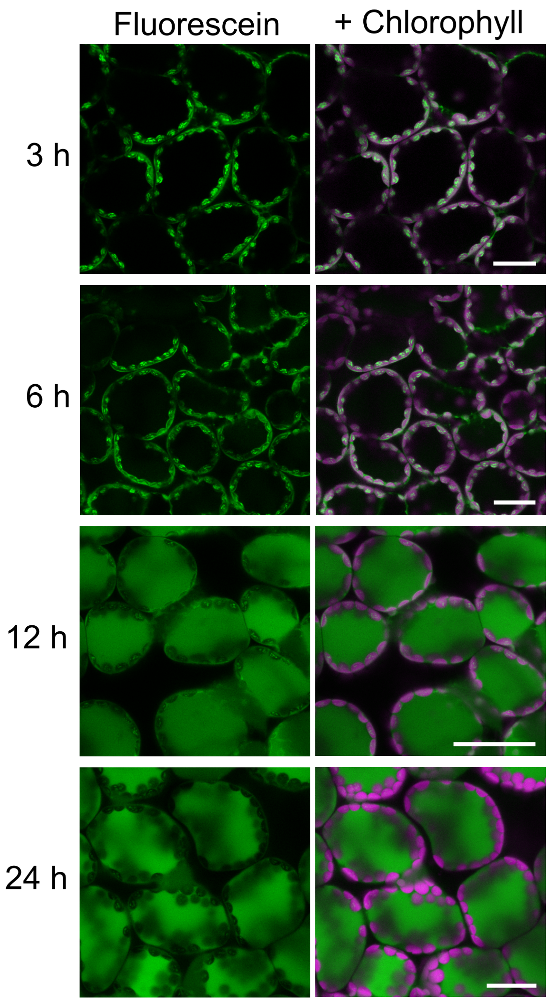

Supplemental Figure S2. Prolonged treatment of fluorescein to Arabidopsis.

Arabidopsis leaves were treated with 10 μM fluorescein for 3, 6, 12, and 24 hours. Scale bars, 25 μm.

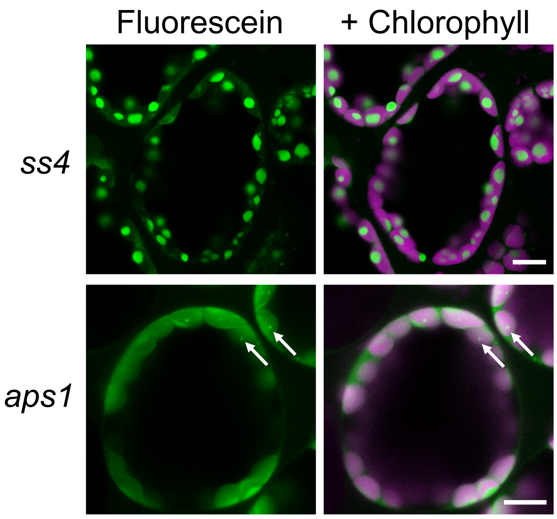

Supplemental Figure S3. Fluorescein allows the visualization of chloroplast starch granules with abnormal number and morphology.

Leaves of Arabidopsis mutants lacking SS4 or APS1 function (*ss4* or *aps1*, respectively) were treated with fluorescein. Arrows indicate tiny cpSGs in the *aps1* mutant. Scale bars, 10 μm.

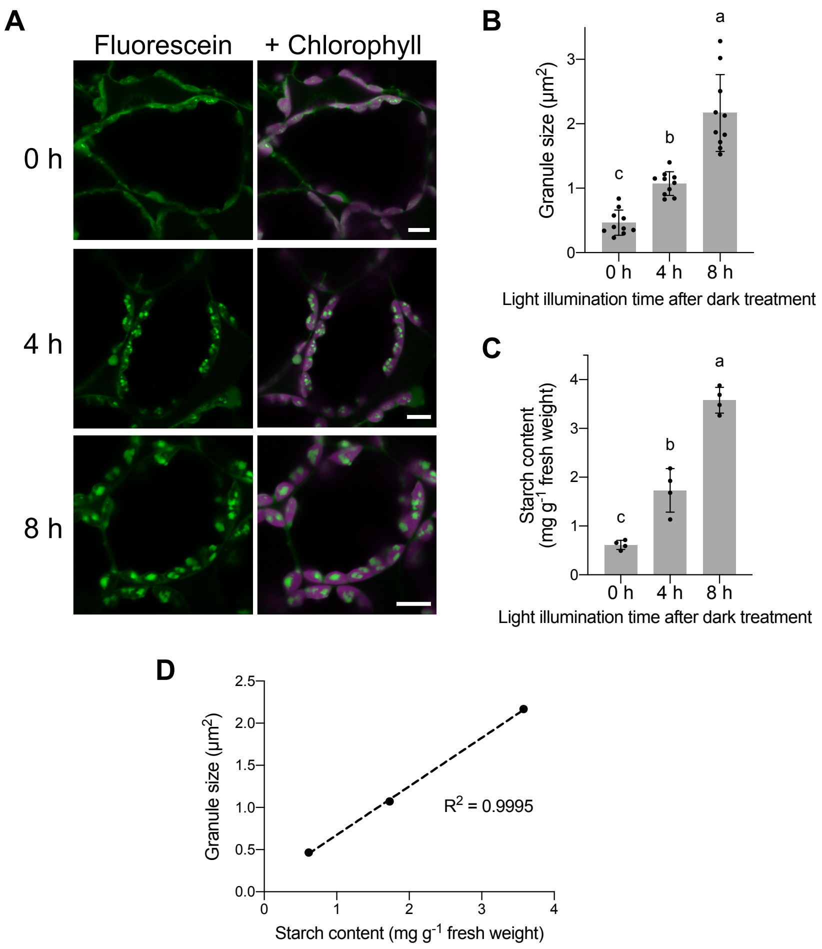

Supplemental Figure S4. Fluorescence signals of fluorescein for chloroplast starch granules correlates with the starch content.

A) Fluorescein-treated leaves exposed to white light (approximately 50 µmol photons m^{-2} s^{-1}) at 22°C for 0, 4, and 8 hours after incubating Arabidopsis under the dark condition for 24 hours. Scale bars, 10 µm. **B)** The size of fluorescent cpSGs at the indicated light-illumination time. Different letters indicate significant differences, as determined using a one-way ANOVA and Tukey's multiple comparison test ($p < 0.05$). Values are means ± SD (n = 10). **C)** The leaf starch content at the indicated light-illumination time. Different letters indicate significant differences, as determined using a one-way ANOVA and Tukey's multiple comparison test ($p < 0.05$). Values are means ± SD (n = 4). **D)** Correlation between the size of fluorescent cpSGs **B)** and the leaf starch content **C)**.

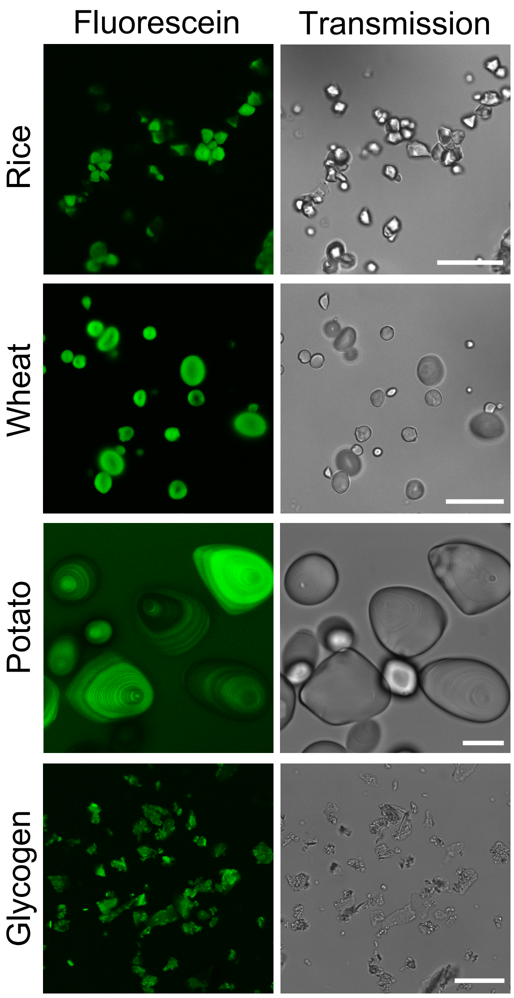

Supplemental Figure S5. Fluorescein stains refined starch.

Refined rice, wheat, and potato starch and glycogen were treated with fluorescein. Scale bars, 25 μm.

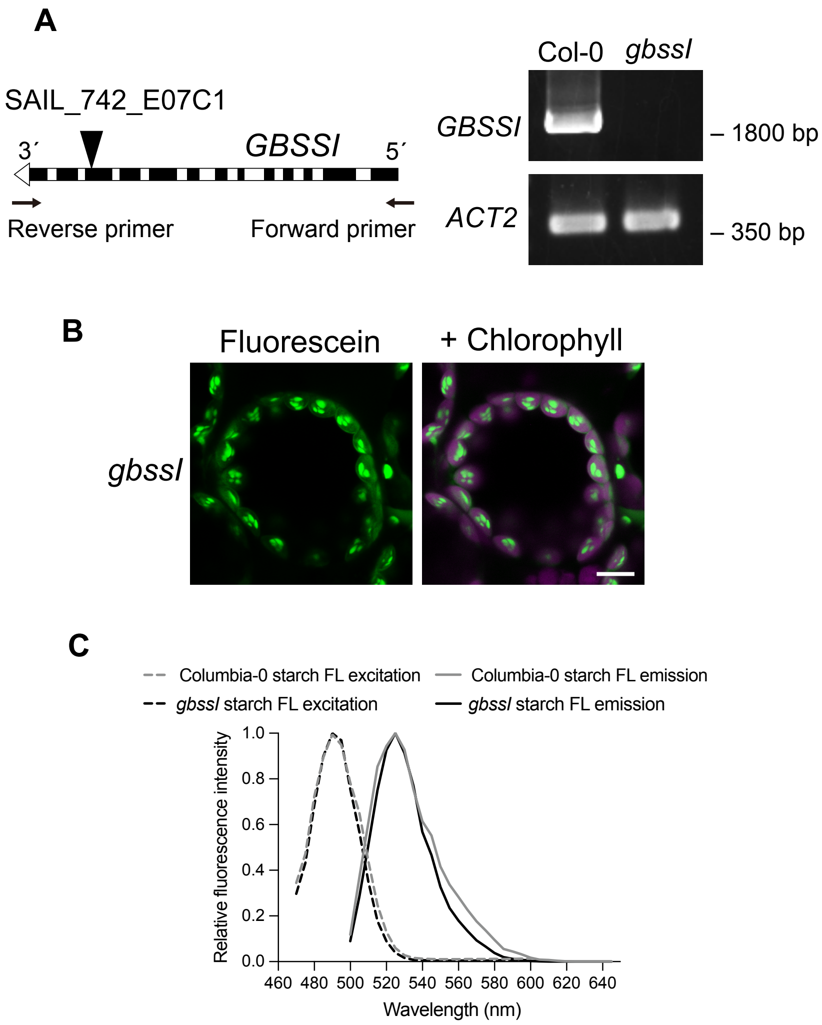

Supplemental Figure S6. Fluorescein stains chloroplast starch granules in the *gbssI* mutant.

A) Characterization of the *gbssI* mutant. In the schematic diagram of the locus, black and white regions indicate exons and introns, respectively. The black arrowhead indicates the T-DNA insertion position within the 11th exon. *GBSSI* expression was not detected in the *gbssI* mutant using RT-PCR. *ACT2* was used as the control gene. **B)** Leaf of the Arabidopsis *gbssI* mutant treated with fluorescein. Scale bar, 10 µm. **C)** The excitation and emission spectra of fluorescein binding to cpSGs from wile type (Columbia-0) or *gbssI* mutant Arabidopsis line measured every 5 nm using xyΛ and xyλ modes, respectively, within LAS X (Leica Microsystems). The measurement was repeated with 10 cpSGs from each Arabidopsis line.

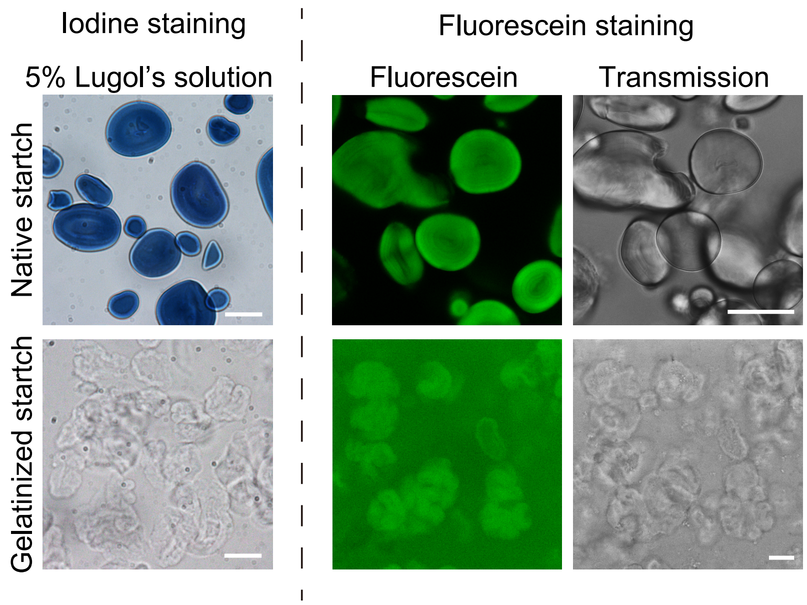

Supplemental Figure S7. Fluorescein stains gelatinized starch.

Wheat starch was boiled in water for iodine staining or the buffer (20 mM HEPES-KOH pH 7.5) for fluorescein staining. Starch gelatinization was confirmed by iodine staining using 5% (v/v) Lugol's solution. Gelatinized wheat starch was treated with 1 μM fluorescein. The gelatinized starch with staining solution was mounted on the 95°C-pretreated slide glass, and the resulting sample was kept at 95°C until observation. Light microscopy (OLYMPUS BX60) with a dry objective lens (UPlanApo 40×/0.85 ∞/0.11-0.23) and confocal microscopy (Leica Microsystems TCS SP8X) with a dry objective lens (HC PL APO 40×/0.95 CORR) were used for observing gelatinized starch stained with iodine and fluorescein, respectively. Scale bars, 25 μm.

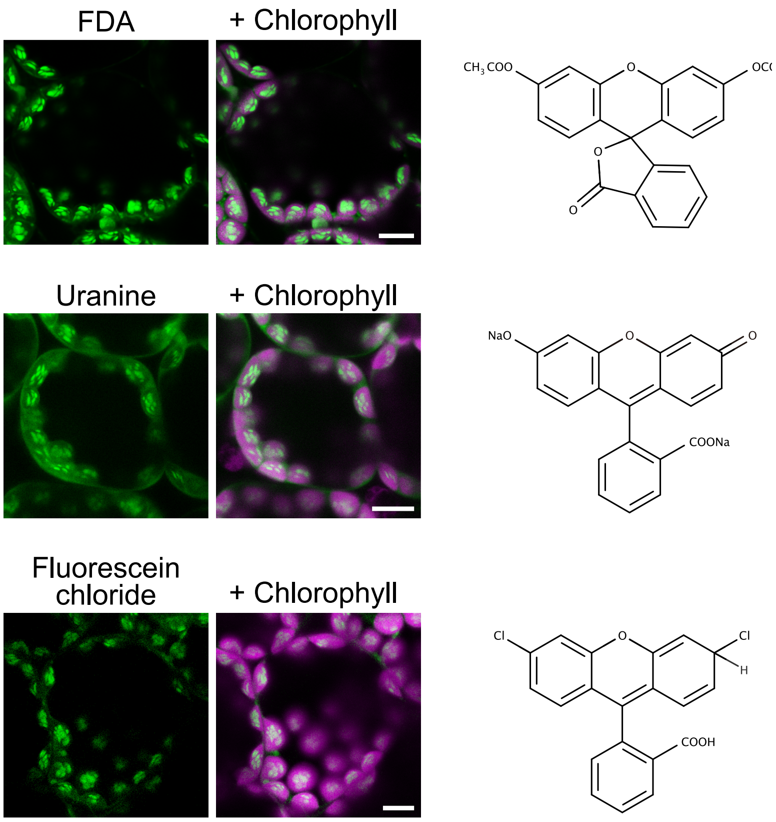

Supplemental Figure S8. Fluorescein derivatives allow the visualization of chloroplast starch granules.

Arabidopsis leaves were treated with fluorescein diacetate (FDA), uranine, or fluorescein chloride. The chemical structures of the dyes are shown at the right of the confocal images. Scale bars, 10 µm.

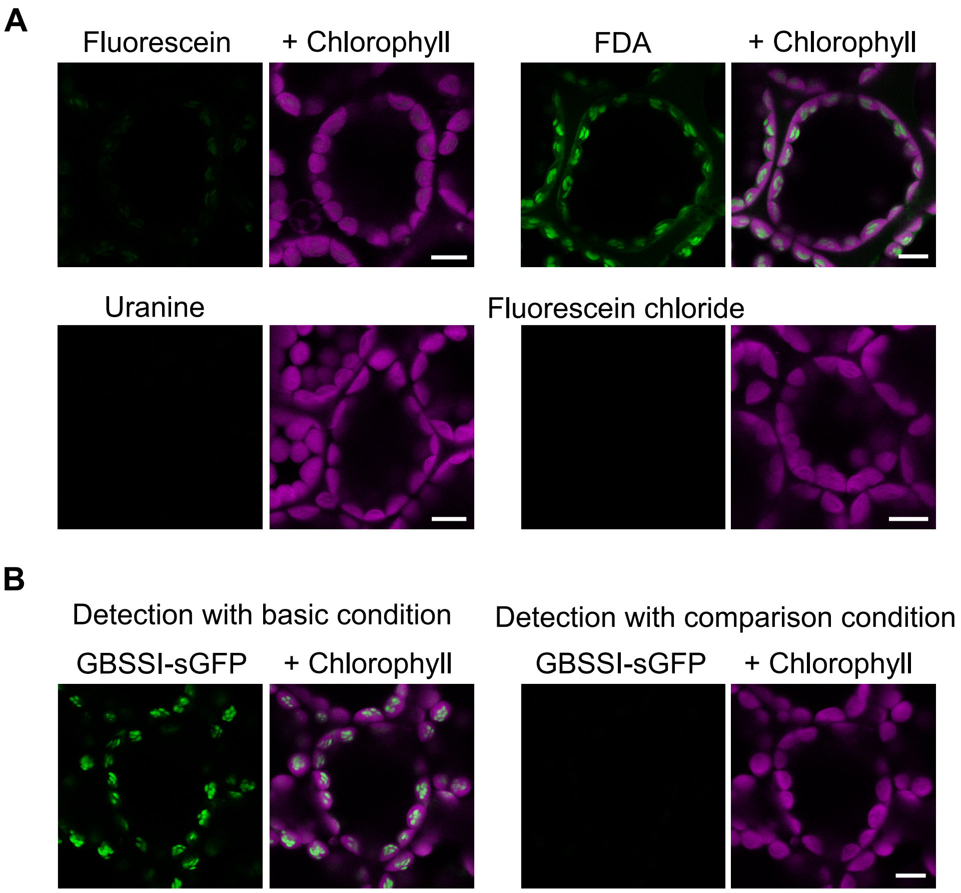

Supplemental Figure S9. Fluorescein diacetate (FDA) is the brightest derivative.

A) Fluorescence of fluorescein and its derivatives (fluorescein diacetate [FDA], uranine, and fluorescein chloride) in cpSGs under fluorescence comparison conditions. Scale bars, 10 µm. **B)** Fluorescence of GBSSI-sGFP in cpSGs under basic and fluorescence comparison conditions. Scale bars, 10 µm.

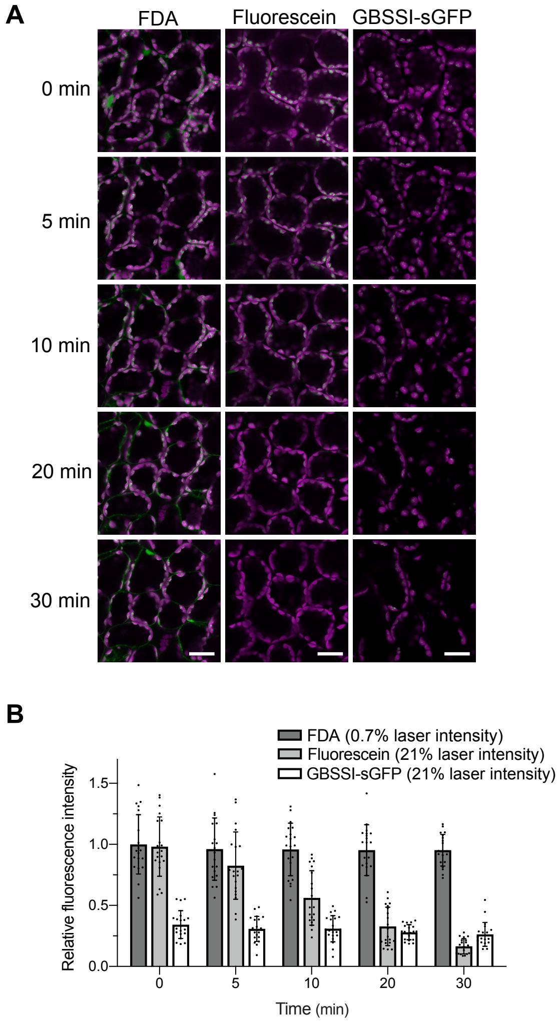

Supplemental Figure S10. Photobleaching test using FDA, fluorescein, and GBSSI-sGFP.

A) Arabidopsis leaves treated with fluorescein diacetate (FDA) or fluorescein and leaves expressing GBSSI-sGFP with continuous laser irradiation for 30 min. To obtain the fluorescence images of cpSGs, 488 nm laser was used for excitation with different laser intensities: FDA with 0.7%, and fluorescein and GBSSI-sGFP with 21%. Fluorescence images were captured at the indicated time and merged with the chlorophyll autofluorescence image. Scale bar, 25 µm. **B)** The relative fluorescence intensity of FDA, fluorescein, and GBSSI-sGFP within the chloroplast at the indicated time. Fluorescence intensity was normalized using the mean value from FDA at 0 min. Values are means ± SD (n = 20).

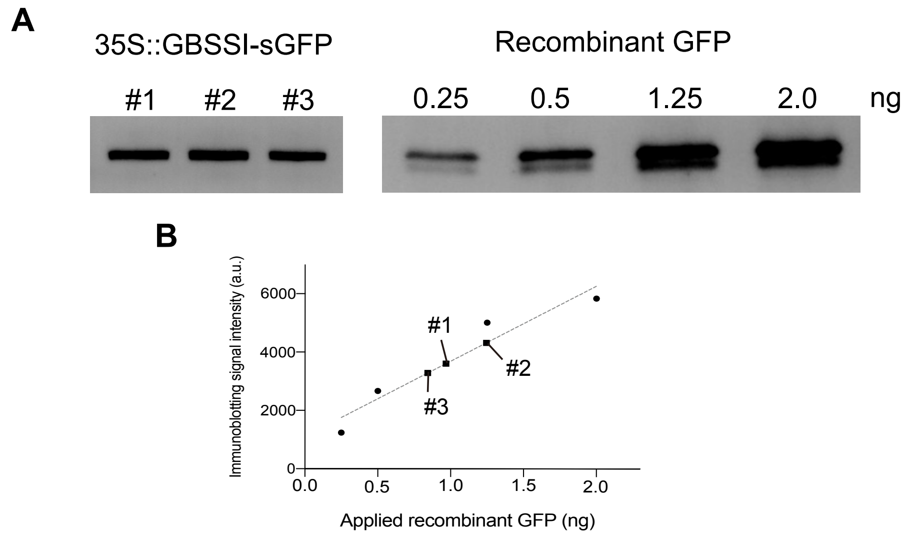

Supplemental Figure S11. Immunoblotting to quantify the abundance of GBSSI-sGFP in transgenic Arabidopsis.

A) Immunoblotting with anti-GFP antibodies. Proteins extracted from the leaves of transgenic Arabidopsis plants expressing *GBSSI-sGFP* were used for immunoblotting. Proteins from three plants of the same transgenic line were loaded as follows: 5,492 ng (sample #1), 5,397 ng (sample #2), and 6,921 ng (sample #3). To determine the abundance of GBSSI-sGFP, recombinant GFP was simultaneously immunoblotted with the above protein extracts. **B)** Standard curve based on the immunoblotting signal of recombinant GFP used to measure the abundance of GBSSI-sGFP. Circular dots indicate the immunoblotting signal of recombinant GFP for the standard curve, and square dots indicate the immunoblotting signal of GBSSI-sGFP. The concentration of GBSSI-sGFP proteins in #1, #2, and #3 was determined to be 0.9677 ng, 1.2449 ng, and 0.8431 ng, respectively. Based on the concentrations of the loading proteins and GBSSI-sGFP proteins, the accumulation of GBSSI-sGFP proteins was estimated to be approximately 0.0176% of total protein in transgenic Arabidopsis.
