# Supplementary material for: Fluorescein staining of chloroplast starch granules in living plants
Source: Plant Physiol. 2023 Oct 4;194(2):662–72. doi: 10.1093/plphys/kiad528 (PMC10828193; doi:10.1093/plphys/kiad528)
Supplement: kiad528_Supplementary_Data [file kiad528_supplementary_data.zip › Supplemental Movie Legends.pdf]

Supplemental Movie S1. The uptake of fluorescein into plant cells.

A deaerated Arabidopsis leaf disc was mounted on a slide glass with 10 µM fluorescein. After mounting the specimen on the slide, we quickly placed the slide glass under the lens of a confocal microscope, set the observation condition to 512×512 pixels with 200-Hz scan speed, and started to observe fluorescein uptake into the cells in the central area of the leaf disc.

Supplemental Movie S2. The rotation movie of the 3D image of rice starch.

This movie was produced using LAS X 3D Viewer (Leica Microsystems).

Supplemental Movie S3. The rotation movie of the 3D image of wheat starch.

This movie was produced using LAS X 3D Viewer (Leica Microsystems).

Supplemental Movie S4. The rotation movie of the 3D image of potato starch.

This movie was produced using LAS X 3D Viewer (Leica Microsystems).

Supplemental Movie S5. The rotation movie of the 3D image of glycogen.

This movie was produced using LAS X 3D Viewer (Leica Microsystems).

Supplemental Movie S6. Three-dimensional illustration of chloroplast starch granules and thylakoids in Arabidopsis mesophyll cells. This movie was produced using Imaris software.
